# Endometrioid adenocarcinoma of the rectovaginal septum with invasion of the rectum: a case report and review of literature

**DOI:** 10.1186/s12957-019-1743-0

**Published:** 2019-12-04

**Authors:** Hua Yang, Jiao-jiao Gu, Yue Qi, Wei Zhao, Xin-lu Wang

**Affiliations:** 1 Department of Ultrasound, Shengjing Hospital of China Medical University, Shenyang, Liaoning Province Republic of China; 2 Department of Obstetrics and Gynecology, Shengjing Hospital of China Medical University, Shenyang, Liaoning Province Republic of China; 3 Department of Pathology, Shengjing Hospital of China Medical University, Shenyang, Liaoning Province Republic of China

**Keywords:** Endometrioid adenocarcinoma, Rectovaginal, Endometriosis, Malignant transformation, Diagnosis

## Abstract

**Background:**

Malignant transformation of endometriosis in the rectovaginal septum is rare and usually misdiagnosed as a colorectal or gynecological tumor. We report a rare case of primary endometrioid adenocarcinoma of the rectovaginal septum with invasion of the rectum.

**Case presentation:**

A 57-year-old overweight woman presented with vaginal bleeding and self-reported left lower abdominal pain during the previous 2 weeks. Preoperative imaging showed a large pelvic mass with invasion of the rectum, suggestive of a gynecologic malignancy. Multiple endoscopic biopsies and immunohistochemical analyses of specimens was performed. The patient received joint gynecological-surgical laparotomy, and there were no intra- or postoperative complications. The histopathological diagnosis was rectovaginal endometrioid adenocarcinoma with rectum infiltration. The patient received adjuvant chemotherapy and achieved good treatment response, with no early complications. At 12 months after surgery, there was no evidence of recurrence.

**Conclusions:**

A high index of clinical suspicion is required for the diagnosis of endometrioid adenocarcinoma in the rectovaginal septum. Surgery combined with additional chemotherapy or radiotherapy seems to be a standard treatment, and hormonal therapy is optional. The efficacies of other therapies, including targeted medication and immunotherapy, are unknown.

## Background

Endometriosis is a chronic and benign gynecological disease most common in women of reproductive age, in which endometrial tissue occurs outside the uterine cavity. The most common locations are the ovaries, fallopian tubes, vagina, broad ligaments, cervix, pouch of Douglas, gastrointestinal tract, rectovaginal septum, and appendix [1, 2]. This disease affects approximately 6 to 10% of women of reproductive age, and the most common symptoms are chronic pelvic inflammation and pain (especially dysmenorrhea) and subfertility [3–5].

Deep-infiltrating endometriosis (DIE) is a sub-class of endometriosis which is defined by endometrial infiltration of the peritoneum by more than 5 mm [6], and endometriosis of the rectovaginal septum is the most severe form [7]. Malignant transformation of endometriosis is quite rare; it occurs in only 0.7 to 1% of patients with endometriosis, and 78.7% of these cases have ovarian malignancies [8, 9]. Primary adenocarcinoma of the rectovaginal septum is extremely rare and most cases are associated with benign endometriosis [10, 11]. In this article, we report a patient with a primary adenocarcinoma arising from endometriosis in the rectovaginal septum with involvement of the rectum.

## Case presentation

A 57-year-old post-menopausal woman (gravida 1, para 1) was admitted to the Gynecology Department of Shengjing Hospital (an affiliate of China Medical University) presenting with vaginal bleeding and left lower abdominal pain for the previous 2 weeks. She had a caesarean section and myomectomy more than 20 years ago and denied any previous hormonal therapy. She was 155 cm in height and 65 kg in weight (body mass index (BMI): 27.1 kg/m^2^). Her mother had a history of pancreatic carcinoma and her father had a history of hepatic carcinoma. She reported no history of weight loss or change in appetite.

The physical and vaginal examination indicated a pelvic mass on the left side with poor mobility. The gynecological examination indicated that the vagina, cervix, and uterus appeared normal. The laboratory tests showed the serum level of CA125 was 207.5 U/mL (normal < 35.0), CA199 was 59.9 U/mL (normal < 37), and HE4 was 206.9 U/mL (normal < 140.0), although the CEA and AFP levels were normal.

**Fig. 1:**
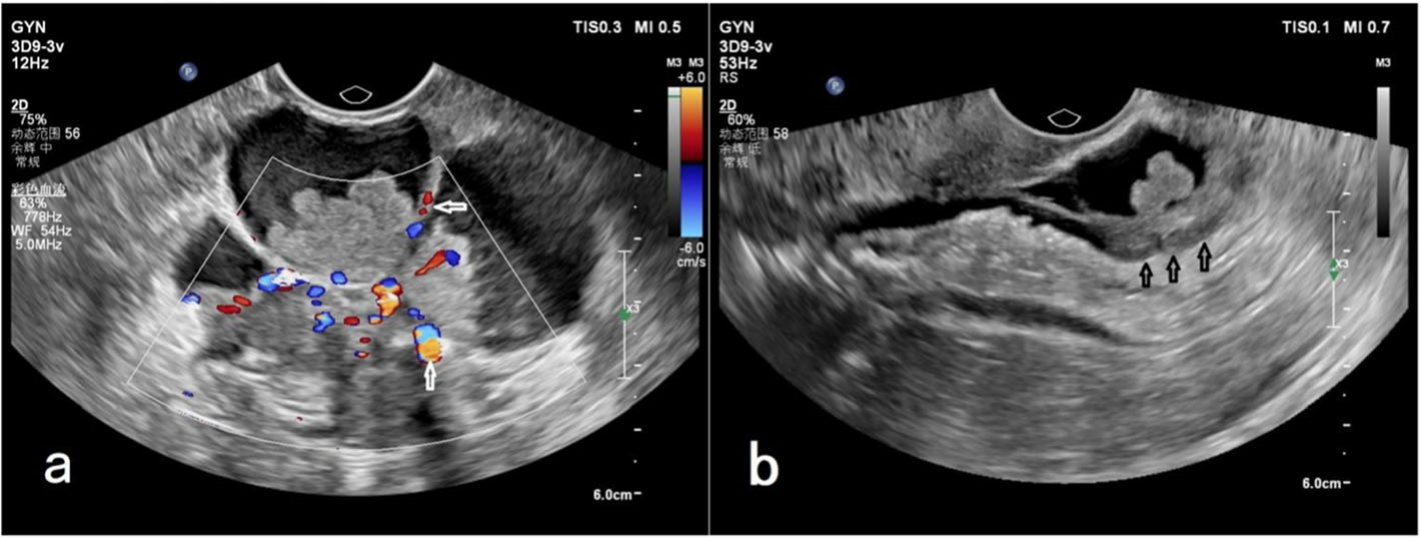
Transvaginal ultrasound (TVA), showing an irregular complex mass in the rectovaginal fossa. **a** Abundant vascularities on the septa and solid portion of the tumor (arrows). **b** Invasion of the mass into the anterior wall of the rectum (arrows)

A transvaginal ultrasound (TVS) showed an irregular complex mass (7.0 × 5.3 × 5.6 cm) in the recto-uterine pouch that had an unremarkable boundary, but had abundant vascularities on the septa and solid portion of the tumor (Fig. 1a). There was also invasion of the mass into the anterior wall of the rectum (Fig. 1b). The right ovary was normal but the left ovary was not visible. A pelvic ultrasound also showed multiple uterine leiomyomas. The endometrium was thickened and irregular, but there was no evidence of ascites, peritoneal implants, or other masses.

**Fig. 2:**
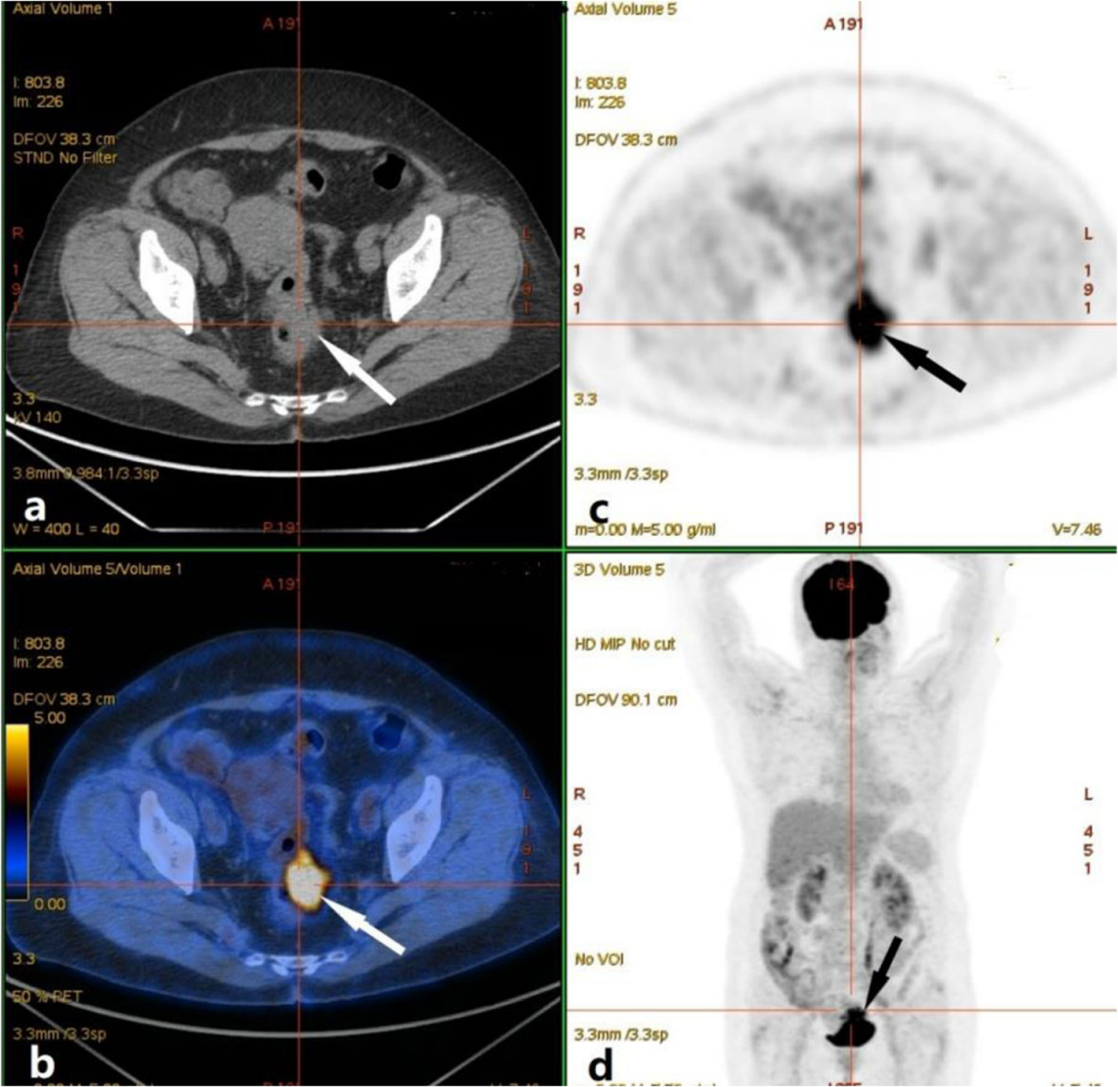
Positron emission tomography/computed tomography (PET/CT), showing a mass with fluorodeoxyglucose (FDG) uptake in the rectovaginal fossa. **a** CT image showing a cystic-solitary mass. **b** PET/CT image showing a mass with FDG uptake (maximum standardized uptake: 11.32). **c**, **d** FDG images

We suspected ovarian carcinoma with invasion of the rectum, and thus performed a whole body FDG-positron emission tomography (PET) (Fig. 2). The F18-fluorodeoxyglucose PET/computed tomography (F18-FDG PET/CT) showed an irregular solid-cystic mass (5.0 × 4.3 cm) in the rectovaginal fossa that had pathologic FDG-uptake and seemed to infiltrate the rectum.

**Fig. 3:**
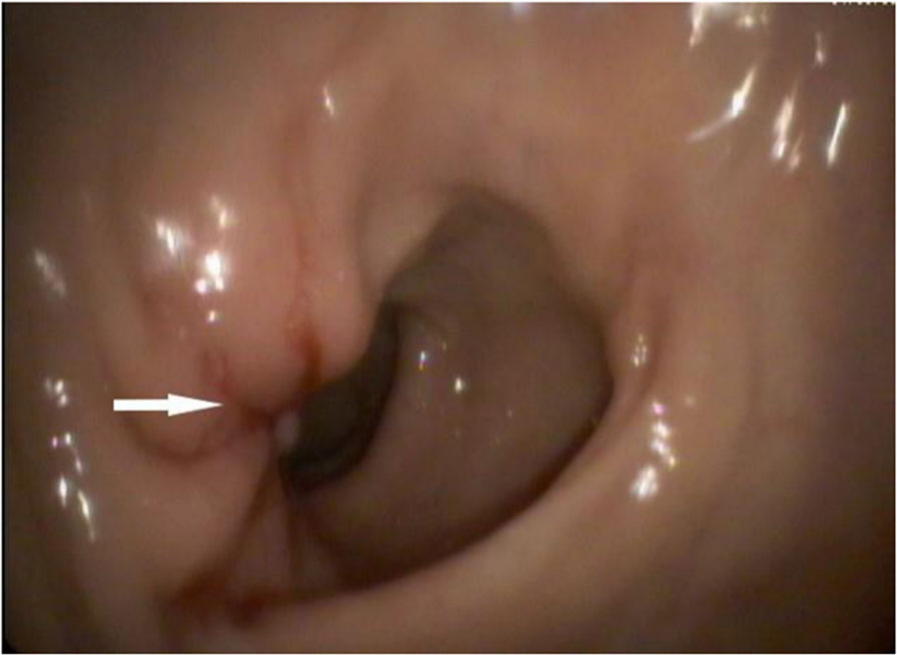
Colonoscopy indicating an ulcerated lesion of the rectum (arrow)

Thus, we also suspected a pelvic malignant tumor infiltrating the rectum. A colonoscopy (Fig. 3) indicated an ulcerated lesion 10 cm from the anal margin that occupied about 1/3 of the lumen, in which the covering mucosa were pale and the surrounding mucosa were clustered. This exam also indicated a 0.5-cm polyp in the sigmoid colon. Multiple endoscopic biopsies were taken during this procedure. An endoscopic ultrasonographic examination indicated a protruding lesion (0.4 × 0.4 cm) in the fundus of the stomach (adjacent to the cardia) and another lesion with fixed echoes in the anterior wall of duodenum (0.4 × 0.4 cm), which was between the third and fourth layers of the duodenal wall. We did not treat these due to their small sizes.

The pathological report of the biopsy specimen indicated evidence of a metastatic adenocarcinoma from a gynecological malignancy with rectum involvement. Thus, a joint gynecological-surgical laparotomy was performed. Radical hysterectomy, bilateral adnexectomy, pelvic peritonectomy, pelvic lymphadenectomy, omentectomy, partial rectal resection with a low pelvic colorectal anastomosis protected by ileostomy, and appendectomy were performed. During this cytoreductive procedure, a 5.0-cm solid mass was identified in the recto-uterine pouch that closely adhered to the rectum and another 3.5-cm cystic mass on the upper rectovaginal septum. Because the mass did not invade the vagina, and considering the patient’s quality of life, an upper vaginal resection with anastomosis was performed, so that the middle and lower segment of the vagina were retained. There were apparent adhesions in the pelvic cavity, especially between the tumors and the rectosigmoid. The bilateral adnexa were atrophic, without any obvious macroscopic tumor. There was no evidence of residual macroscopic lesions or intraoperative complications after surgery.

**Fig. 4:**
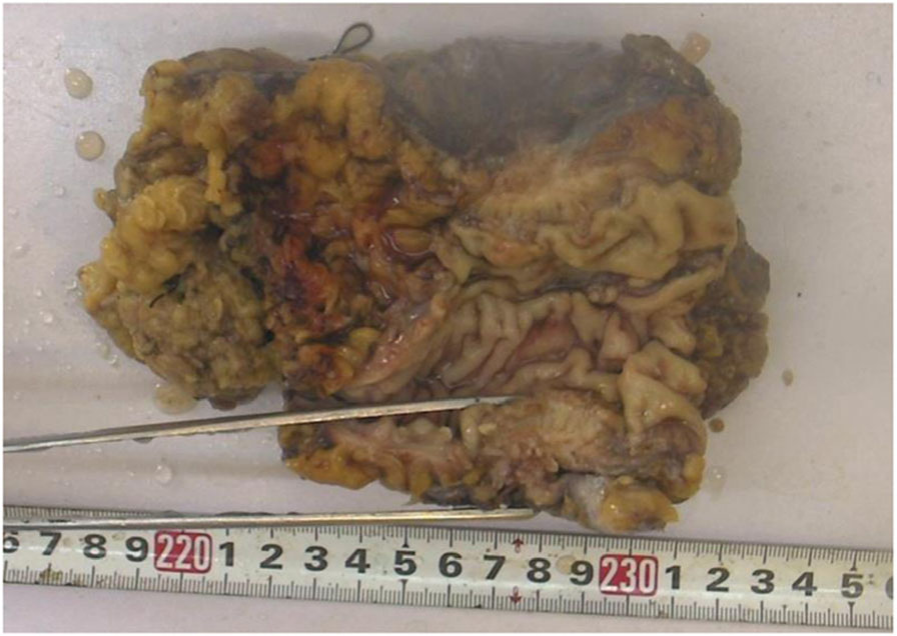
Section of the rectal specimen indicating a thickened wall

**Fig. 5:**
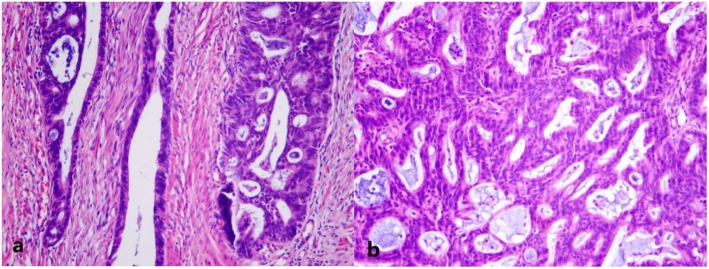
Histopathological findings (hematoxylin-eosin (H&E) staining, 100×). **a** Adenocarcinoma invasion of the rectum. **b** Solid part of the tumor, indicating heteromorphic cells arranged in a sieve pattern

**Fig. 6:**
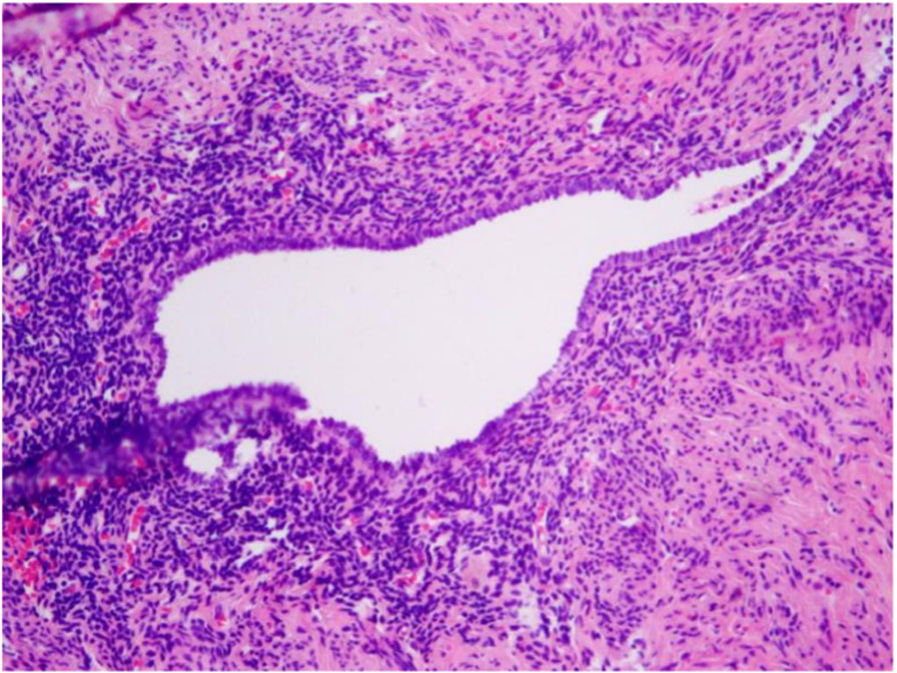
Histopathological findings (H&E staining, 100×), showing endometriosis in the left adnexa

Analysis of the resected specimens indicated the rectal specimen had a thickened wall with coarse mucosa and serosa (Fig. 4). When opening the cystic mass adhering to the rectum, the internal wall was gray-yellow in color with cauliflower-like lesions. For histological analysis, specimens were fixed in 10% buffered formalin, processed, and embedded in paraffin. Microscopic analysis indicated the tumor cells were acinar, with papillary structures that were moderately differentiated, and heteromorphic cells were arranged in a sieve pattern (Fig. 5). Endometriosis was also evident in the left adnexa (Fig. 6).

**Fig. 7:**
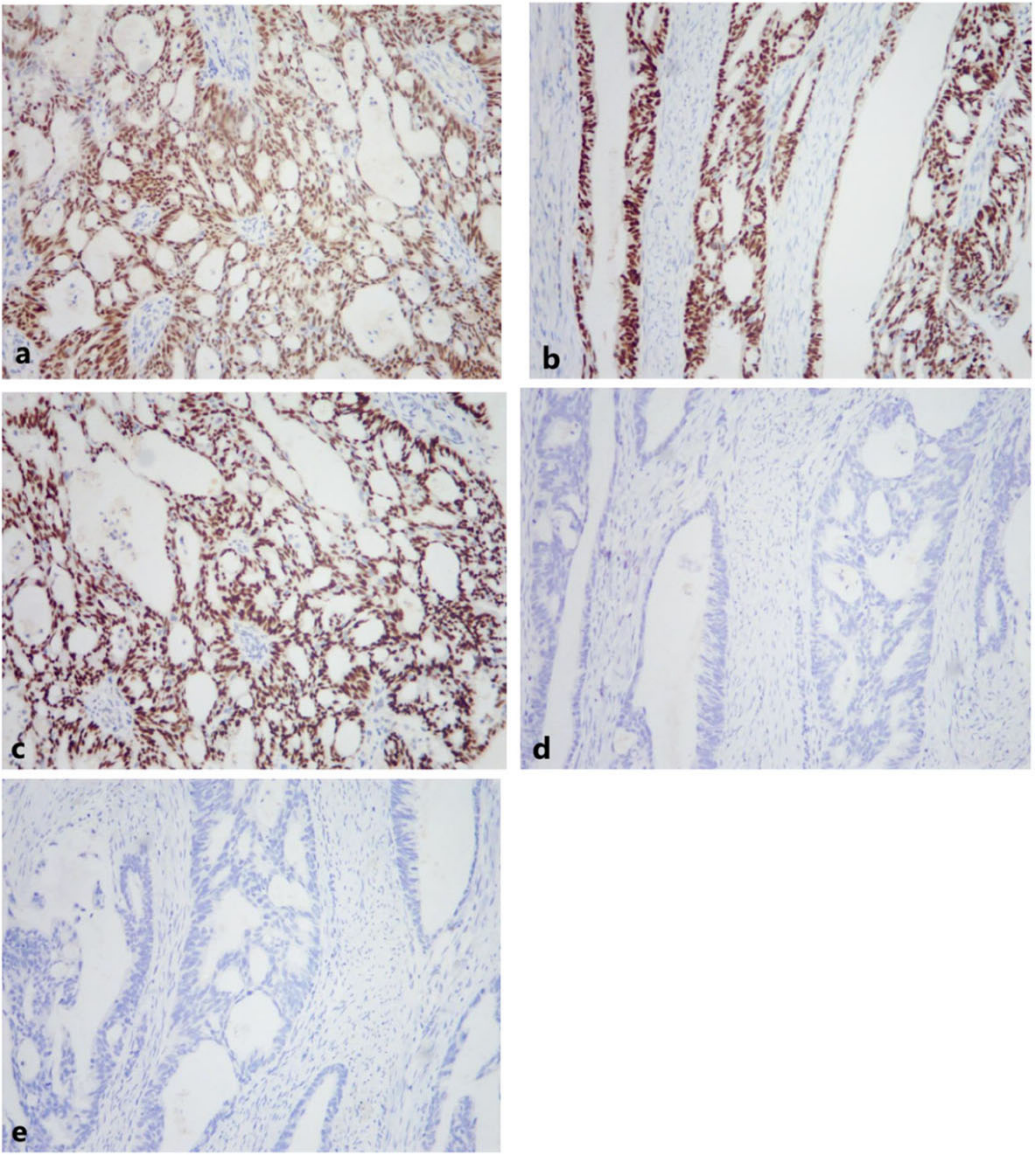
Immunohistochemical (IHC) staining of tumor cells for 5 markers (100×). **a** PAX-8 (positive). **b** ER (positive). **c** CK7 (positive). **d** CK20 (negative). **e** CDX-2 (negative)

Immunohistochemical studies performed using the avidin-biotin peroxidase complex technique indicated positive staining for cytokeratin 7 (CK7), estrogen receptor (ER), paired box gene 8 (PAX-8), and Wilms tumor protein (WT-1), weakly positive staining for progesterone receptor (PR), and no staining for CK20, caudal-related homeobox 2 (CDX-2), vimentin, P53 proteins, and villin (Fig. 7). In addition, 10% of the cells were positive for Ki-67 (data not shown). The final histopathological diagnosis was rectovaginal endometrioid adenocarcinoma with rectum infiltration. Thus, the patient received 6-month adjuvant chemotherapy and achieved good treatment response. The patient has been free of disease for 12 months since the surgery. A follow-up at that time, which included enhanced CT and TVS, showed no evidence of recurrence.

## Discussion and conclusions

Endometriosis is a common gynecological disease among premenopausal women that is characterized by uncontrolled endometrial cell proliferation, with local and distant spread of these cells [12]. Although endometriosis is not considered a premalignant disease, it may nonetheless have malignant potential and aggressive pathology, characterized by high local invasiveness and recurrence [13]. In 1925, Sampson [14] first described malignant transformation in a patient with endometriosis. This condition may occur in 0.7 to 1% of women with endometriosis [15]; it is most common in the ovary, but about 20% of cases have malignancies at extragonadal sites [16]. Sampson [14] and Scott [17] proposed four basic criteria for diagnosis of endometriosis-associated cancer: (i) malignant and benign endometrial cells coexist within the same tissue; (ii) the malignancy originates from the same tissue, without metastasis or infiltration from other sites; (iii) there are no other primary sites; and (iv) the adjacent endometriosis focus is contiguous with the endometrioid carcinoma tissue. Our patient fulfilled all four criteria.

The rectovaginal septum is a relatively common site for extragonadal endometriosis, followed by the ovary. Although endometriosis-associated rectovaginal adenocarcinoma is extremely rare, it accounts for 70% of primary rectovaginal malignancies [18, 19]. A recent comprehensive review [19] found reports of fewer than 20 primary carcinomas of the rectovaginal septum arising from endometriosis. We identified 8 full-text articles published in English between 1950 and 2018 that described 9 patients with primary endometriosis-associated carcinoma (Table 1). As expected, adenocarcinoma is the most common histologic type of extragonadal endometriosis-associated neoplasm (6 cases including the present patient). The less common histologic forms were clear cell carcinoma, stroma sarcoma, adenoacanthoma, and carcinosarcoma [20].

**Table 1:** Characteristics of our patient and previously reported patients with rectovaginal septum tumors related to endometriosis

Author/year	Patient age (years)	Signs/symptoms	Medical history	Body type	Laboratory tests	Radiology/ultrasonic findings	Histology	Treatment	Follow-up
Dockerty et al. [1], 1954	54	Serosanguineous vaginal discharge	Thyroidectomy	ND	ND	ND	Adenocarcinoma	TH+BSO+LN/RT	DOD 2 years
Dockerty et al. [1], 1954	45	A small reddish area on the posterior lip of the cervix	ND	Obese	Normal	ND	Adenocarcinoma	TH+BSO+LN/RT	NR 10 years
Lash and Rubenstone [2], 1959	32	Severe low back pain, cyclic vaginal bleeding	STH	Obese	Normal	Upper and lower gastrointestinal roentgen studies were normal	Adenocarcinoma	Cervicectomy, RR	ND
Young and Gamble [3], 1969	47	Intermittent vaginal bleeding, pelvic pain, and a cul-de-sac mass	STH	ND	ND	ND	Adenoacanthoma	Pelvic exenteration+RT	Unknown
Goldberg et al. [4], 1978	48	A hemorrhagic nodule on the posterior vaginal wall	Spontaneously aborted through a laceration	ND	ND	ND	Clear cell adenocarcinoma	TH+LN+RR+resection of the upper half of the vagina	Metastatic nodes 9 months later
Addison et al. [5], 1979	37	Vagina1 and rectal bleeding	TH+celiotomy+nephrectomy	Obese	ND	ND	Adenoacanthoma	RT/CT	DOD 1 year
Yazbeck et al. [6], 2005	25	Lower abdominal pain and dyspareunia; painful retrocervical nodule	Total thyroidectomy + appendectomy	ND	CA125: 700 U/mL	US showed a heterogeneous pelvic mass; MRI confirmed the central pelvic mass.	Papillary adenocarcinoma	RT/TH+RR	NR 2 years
Ulrich et al. [7], 2005	51	Irregular vaginal bleeding	Vaginal hysterectomy	ND	ND	Pelvic MRI confirmed a tumor of the rectosigmoid colon	Glandular and papillary tumor	RR+BSO+vagina and parakolpium resection+LN+RT	RE 2 years later
Mabrouk et al. [8], 2011	36	Abdominal discomfort	Unknown	ND	Ca125 and Ca19.9 were elevated	CT scan showed a retro-uterine mass; US scan revealed both slightly enlarged ovaries and a retrocervical mass	Clear cell and endometrioid adenocarcinoma	TH+LN+omentectomy+appendicectomy+CT(cisplatinum)+RR□	NR 2 months
Present case, 2019	57	Vaginal bleeding and left lower abdominal pain	Caesarean section and myomectomy	Overweight	Ca125, Ca19.9, and HE4 were elevated	US scan showed an irregular complex mass in the rectovaginal fossa, PET/CT showed a mass with FDG uptake in the rectovaginal fossa.	Adenocarcinoma	TH+LN+omentectomy+peritonectomy+appendicectomy+partial rectal resection+CT	NR 6 months

*RT*, radiation therapy; *TH*, total hysterectomy; *STH*, subtotal hysterectomy; *BSO*, bilateral salpingo-oophorectomy; *LN*, lymph node dissection; *CT*, chemotherapy; *RR*, rectal resection; *RE*, recurrence; *NR*, no recurrence; *DOD*, dead of disease; *ND*, not described; *US*, ultrasound; *MRI*, magnetic resonance imaging; PET/CT, positron emission tomography/computed tomography; *FDG*, fluorodeoxyglucose

The etiology of rectovaginal endometriosis remains uncertain. One possible mechanism is the adhesion and growth of endometrial tissues that were deposited into the peritoneal cavity via retrograde menstruation. It is also possible that the origin is from metaplasia of the Müllerian remnants in the rectovaginal septum [21]. Endometriosis is an estrogen-dependent disease, and some evidence indicated that endogenous or exogenous hyperestrogenism may contribute to the malignant transformation [9]. Young et al. [22] reported a patient with rectovaginal endometriosis-associated adenocarcinoma who was taking high-dose unopposed estrogens (1.25 mg conjugated estrogens per day) for 14 years after a subtotal hysterectomy. On the other hand, obesity also increases the risk for developing cancer from endometriosis. Three of the 8 women with rectovaginal tumors arising from endometriosis were obese (Table 1) [23, 24]. Our patient did not use unopposed estrogens, but her BMI was above normal. It is also interesting to note that 6 of the 10 (60%) cases we reviewed (including our patient) had previous lower abdominal/pelvic surgery, including hysterectomy. Thus, pelvic surgery itself could increase the risk for dissemination of endometriotic lesions and malignant transformation of rectovaginal endometriosis. Okimura et al. [8] also mentioned this possibility.

The average patient age was 43.2 years. Our patient was 57 years old, older than any of the other 9 patients. Women with rectovaginal endometriosis–associated adenocarcinoma often complain of dyschezia, deep dyspareunia, abdominal or pelvic pain, and vaginal or rectal bleeding. Our patient’s chief complaints were vaginal bleeding and left lower abdominal pain. In fact, due to the deep location of these tumors and the atypical clinical symptoms, diagnosis is often difficult, so these tumors often remain latent for a long time before diagnosis. In the present case, the tumor was already more than 5 cm in diameter, and it had infiltrated the entire wall of the rectum upon diagnosis. In all cases, diagnosis requires surgery and pathologic examination.

For the very few cases of previously reported rectovaginal septum tumors arising from endometriosis, modern imaging techniques were usually used for initial evaluation of local and regional extension of the tumor, but were insufficient for diagnosis because the results are also consistent with uterine or ovarian tumors, just as in our patient. Transvaginal ultrasound (TVS) is a first-line technique used to examine a pelvic mass and DIE. In particular, TVS can evaluate the exact location and extent of infiltration, which is important for determining the type and difficulty of surgery [25]. For instance, TVS of our patient showed a large mass in the recto-uterine pouch with rectal wall infiltration. Moreover, magnetic resonance imaging (MRI) can also help to evaluate deep lesions in the rectovaginal region. A meta-analysis by Guerriero et al. [4] demonstrated similar diagnostic performance of TVS and MRI in the detection of DIE, confirming the role of TVS as a cost-effective first-line technique. Nevertheless, preoperative imaging techniques are only useful for estimation of the location and extent of infiltration, and cannot be used to determine malignant transformation of endometriosis. When rectum infiltration is suspected, diagnostic rectoscopy or colonoscopy should be performed, and endoscopic biopsies may also be needed for pathological examination. In our patient, pathological analysis of the colonoscopy biopsy specimen indicated a metastatic adenocarcinoma from a gynecological malignancy.

After biopsy or resection of the tumor, immunohistochemical staining (IHC) may be essential to confirm the diagnosis. IHC staining using antibodies against ER, PR, CK7, PAX-8, CK20, and CDX2 is extremely useful for the differential diagnosis of endometrioid adenocarcinoma and primary intestinal adenocarcinoma. In particular, IHC staining for CK7 and CK20 can differentiate endometrioid and primary rectal carcinoma [26]. Many colorectal carcinomas are CK20-positive and CK7-negative, but gynecological tumors (including endometrioid carcinomas) are often CK20-negative and CK7-positive. PAX-8 is a specific marker of Müllerian origin and is also expressed in gynecological tumors. Furthermore, the ER is expressed in most uterine endometrioid adenocarcinomas. On the other hand, CDX2 (a homeobox transcription factor) is expressed during normal intestinal development [27]. Thus, CDX2 expression may be positive in colorectal cancers and has high sensitivity for the differential diagnosis of colorectal adenocarcinoma [28]. Our patient had positive IHC staining for CK7, ER, and PAX-8, and negative staining for CK20 and CDX-2, compatible with our diagnosis of endometrioid adenocarcinoma.

There is currently no consensus on the standard therapeutic approach to be used for treating extraovarian endometriosis–associated malignancies, and studies in the literature report the use of highly individualized treatments. However, primary surgical excision or radical resection of the tumor should be performed if feasible. Including our patient, 9 of 10 cases received surgery (Table 1). Among the previous 9 cases, 7 received total hysterectomy and/or salpingo-oophorectomy and 2 received local resection. Moreover, 6 patients received rectal resections and 6 patients received lymph node dissections. Some clinicians have offered adjuvant therapy, including chemotherapy and radiotherapy. Therefore, chemotherapy may be the first-line adjuvant treatment for aggressive malignant transformation of extragonadal endometriosis [29]. Similar to treatments for endometrial cancer, the chemotherapeutic regimens typically consist of platinum-taxane combinations. The therapeutic value of chemotherapy for extragonadal endometriosis–associated cancer is unclear. Radiation therapy may be performed after surgery or for treatment of local recurrence after the primary surgery and chemotherapy. Because of the rarity of this disease, neoadjuvant therapy should be considered, as should hormone-therapy for gestagen receptor-positive endometriosis-associated cancers [30]. Primary endometrioid adenocarcinoma of the rectovaginal septum has much better prognosis than endometrioid tumors at other sites [11].

In conclusion, endometrioid adenocarcinoma of the rectovaginal septum is a rare malignant tumor that requires a high index of clinical suspicion for successful diagnosis. Clinicians must differentiate this tumor from colorectal cancers so that the most appropriate treatment is administered. Because of the rarity of this tumor, we cannot draw any conclusions regarding preoperative diagnosis. We suggest clinical suspicion in patients previously diagnosed with endometriosis who present with abdominal pain and vaginal or rectal bleeding, especially in women taking unopposed estrogens. Several imaging techniques can help evaluate local and regional extension of the tumor. IHC staining is essential for the final diagnosis. Surgery combined with chemotherapy or radiotherapy seems to be the standard treatment for rectovaginal malignancies arising from endometriosis, but hormonal therapy could be considered depending on the individual patient. However, because of the rarity of this condition, identification and characterization of additional patients is essential. The application of targeted medication and immunotherapy may help to improve prognosis.

## Data Availability

The datasets generated and analyzed during the present study are available from the corresponding author on reasonable request.

## Abbreviations

AFP: Alpha-fetoprotein
BMI: Body mass index
CA125: Cancer antigen 125
CA19-9: Cancer antigen 19-9
CEA: Carcinoembryonic protein antigen
CDX-2: Caudal-related homeobox 2
CK7: Cytokeratin 7
CK20: Cytokeratin 20
DIE: Deep-infiltrating endometriosis
F18-FDG PET/CT: F18-fluorodeoxyglucose PET/computed tomography
ER: Estrogen receptor
HE4: Human epididymis secretory protein 4
IHC: Immunohistochemical
MRI: Magnetic resonance imaging
PAX-8: Paired box gene 8
PR: Progesterone receptor
TVS: Transvaginal ultrasound
WTP-1: Wilms tumor protein-1
